# Observed data quality concerns involving low-cost air sensors

**DOI:** 10.1016/j.aeaoa.2019.100034

**Published:** 2019-07-01

**Authors:** Andrea L. Clements, Stephen Reece, Teri Conner, Ron Williams

**Affiliations:** a U.S. Environmental Protection Agency, Office of Research and Development, Research Triangle Park, NC, 27711, USA; b Oak Ridge National Laboratory, Oak Ridge, TN, 37831, USA

**Keywords:** Low-cost sensor, PM_2.5_, tVOC

The US EPA’s emerging technologies research program has been actively evaluating the performance of low-cost (< $2500 USD) air quality sensors (Evaluation of Emerging Ai). This work carefully documents the performance of this technology class versus regulatory-grade instrumentation and communicates the findings to a wide variety of stakeholders via a publicly-accessible web portal. While not defining the potential value of any given commercially-available sensor for any given application, key parameters such as accuracy, precision, and other response characteristics are reported. There are currently no regulatory or manufacturer’s requirements regarding performance metrics of these products (Woodall et al., 2017), and unlike more costly regulatory instruments, low-cost sensors often do not provide a means for calibration, flow check, or quality control procedures that can help maintain performance.

Increased availability of commercially-available low-cost air quality sensors combined with increased interest in their use by citizen scientists, community groups, and professionals has resulted in a flood of journal articles describing environmental findings. These new technologies offer an unprecedented ability to measure air quality at denser spatial scales, shorter temporal scales, and under a variety of conditions (e.g., mobile). Even so, an extensive review of recent peer reviewed journal articles indicated data quality issues (e.g., variable performance within a batch of sensors, handling of outliers, measurement artifacts) were rarely being investigated, defined, or reported on as part of published findings (Williams et al., 2018). This is quite alarming considering air quality has historically been well characterized and professionals have used well-known best practices to perform quality control checks and sometimes carefully exclude data from analyses when appropriate.

We have reported examples of poor performance characteristics in our evaluations including co-responsiveness to high relative humidity for nephelometric-based particulate matter (PM) sensors or low temperature effects on electrochemical gas phase sensors (Feinberg et al., 2018; Williams et al., 2014). Such effects have often been easy to discover or define. We share here two examples of less obvious performance characteristics involving the response of a commercially-available low-cost PM sensor and a tVOC (total volatile organic compound) sensor. While the commercial identities of the sensors are not reported here, their response issues are indicative of the type of data quality investigations we urge researchers to consider when validating data for publication or other professional purposes.

The two sensor types reported here were deployed consistent with the manufacturer’s suggested guidelines with minimal exception. In particular, the tVOC sensor has a stated RH limit of 90%. Ambient conditions sometimes exceeded that value and, as shown in the findings, resulted in the sensor being corresponsive to this parameter. The tVOC sensor was described as having minimal response error associated with temperatures from −20 to 60 °C. The PM sensor listed an operation range between 1 and 99% RH and −20 to 60 °C.

The sensor performance describes “new” or “out-of-the-box” performance. Both sensor types were tested for basic operating functionality prior to deployment. The tVOC sensor was satisfactorily tested for a linear response to a mix of gaseous challenge compounds in a controlled laboratory setting prior to its use along with 9 other units and found to be fully operational. Both sensors were deployed in small batches (3 + units) under ambient conditions to determine if response was generally consistent among identical units. The findings reported here represent a sensor-to-sensor comparison, not a direct comparison with collocated reference monitors. Generally, the agreement between identical sensors is higher than the sensor to reference comparison and thus, these findings represent a “best-case” indicator of performance.

Fig. 1 compares data from two identical PM sensors reporting collocated measurements over a period of approximately 100 days. Sensor were regularly check and no operational issues were observed. Further, no error messages or data flags were reported. Two distinct response curves are apparent (with a potential third emerging) representing a sudden shift in the response (offset of approximately 25 μg/m^3^) for this nephelometric-based PM sensor approximately 60 days into the evaluation period. In this case, some of the more obvious influences (e.g., relative humidity) were not associated with the shift in response. Factors such as changing internal sensor conditions (e.g., lamp intensity, fan speed, cleanliness of optics) or even variations in the minimum particle size detected (which could increase uncertainty when the particle size distribution, PM morphology, or dominating aerosol sources change) might be responsible. In this example (Fig. 1), nucleation events did not influence the behavior illustrated. The observed shift happened more than 30 days after the beginning of the deployment, so a short-term collocation would not have observed the phenomenon. Additionally, a pre-and post-deployment collocation could not identify when the shift occurred or if there was more than one. As a result, the concentration reported by this sensor is highly uncertain and relative difference between identical sensors is also unreliable. Users attempting to use data from such devices without understanding this response duality could easily over or underestimate local air quality concentration estimates or lead one to attribute concentration variations inappropriately.

A second example documents the impact of environmental factors (relative humidity and temperature) on a popular low-cost tVOC photoizonization detector (PID) operating in an ambient environment during a 1-week collocation period. The PID is a non-specific VOC sensor and co-responsive to a wide range of organics. In Fig. 2, the response curves of four collocated sensors are shown. The data markers are colored by the percent relative humidity. When these sensors experience low temperatures (< 25 °C) and high relative humidity (> 75%), the set of sensors begin exhibiting inconsistent response (instantaneous differences of > 100 ppb at times). As similar temperature and RH conditions are routinely encountered across many geographical areas, understanding the unpredictability of the response is important. While changing tVOC concentration and composition could be influencing the overall response of the PID, the loss of correlation between collocated units dropped dramatically as RH increased as shown in Fig. 3 (r ≤ 0.27 at RH ≥ 60%). The observation that replicate PID units operating under the same environmental conditions might yield vastly different response relationships under certain meteorological conditions represents a challenge to their successful use for research purposes. These findings also show that deployment of these sensors in environmental conditions where RH > 90%, as is often encountered in rain and/or coastal conditions, might easily result in a response unrelated to the true tVOC concentration.

These examples highlight the caution we urge the scientific community to take in the use and reporting of low-cost sensor data. At a minimum, performance of multiple sensors over the range of environmental conditions experienced must be investigated and repeated collocation and/or continuous comparison is important for identifying shifts in response or aging. Even more importantly, citizen scientists and other non-professionals attempting to use such data and potentially not having the knowledge needed to carefully screen out poor or questionable data need to be aware of these issues. Regulatory officials and others being challenged with data from such sources must be cautious in how such data might be used in local air quality decision making.

## Figures and Tables

**Fig. 1.**
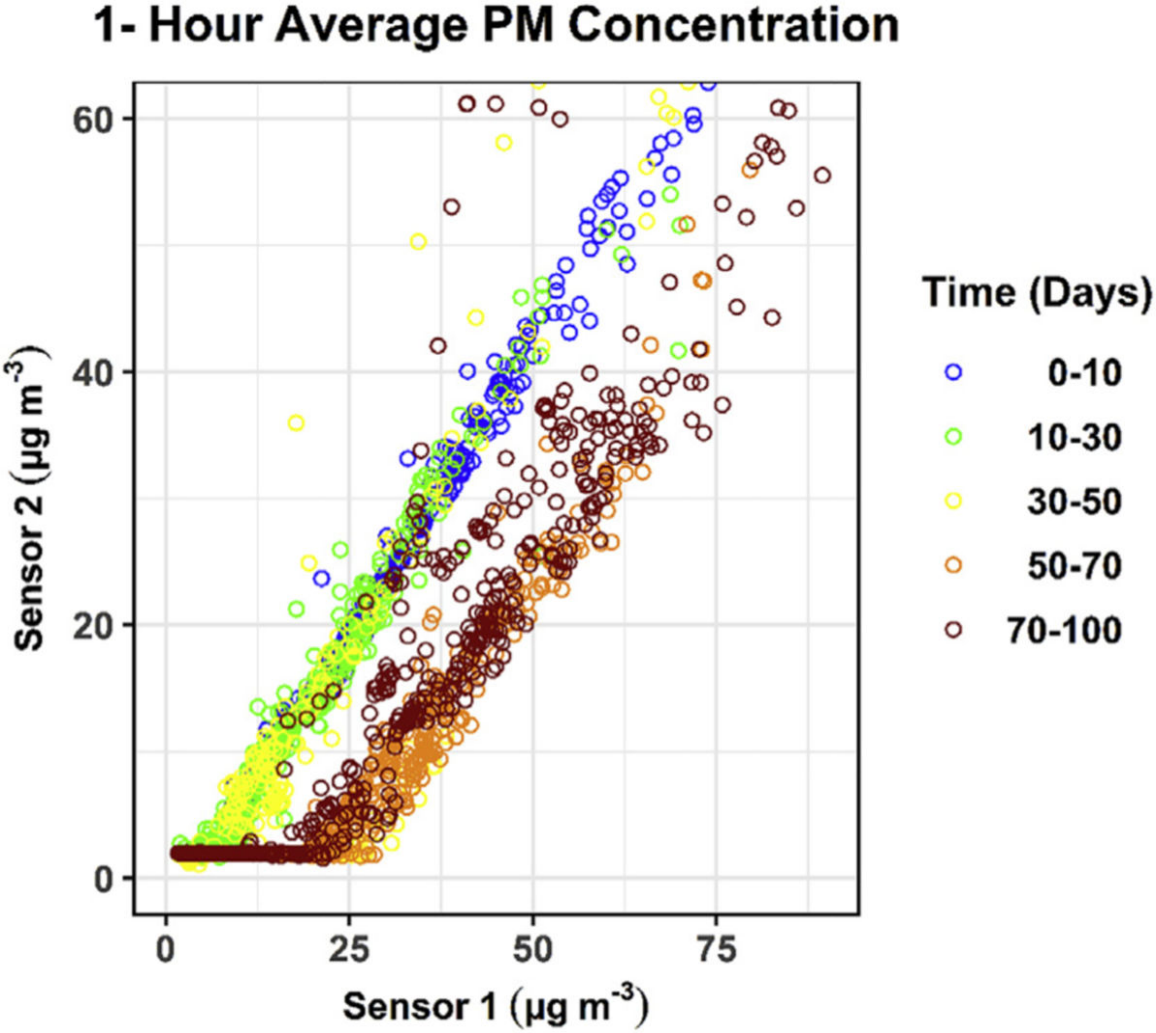
Deployment time-dependent response of replicate collocated nephelometer-based low-cost PM sensors showing the response impact associated with the length of deployment.

**Fig. 2.**
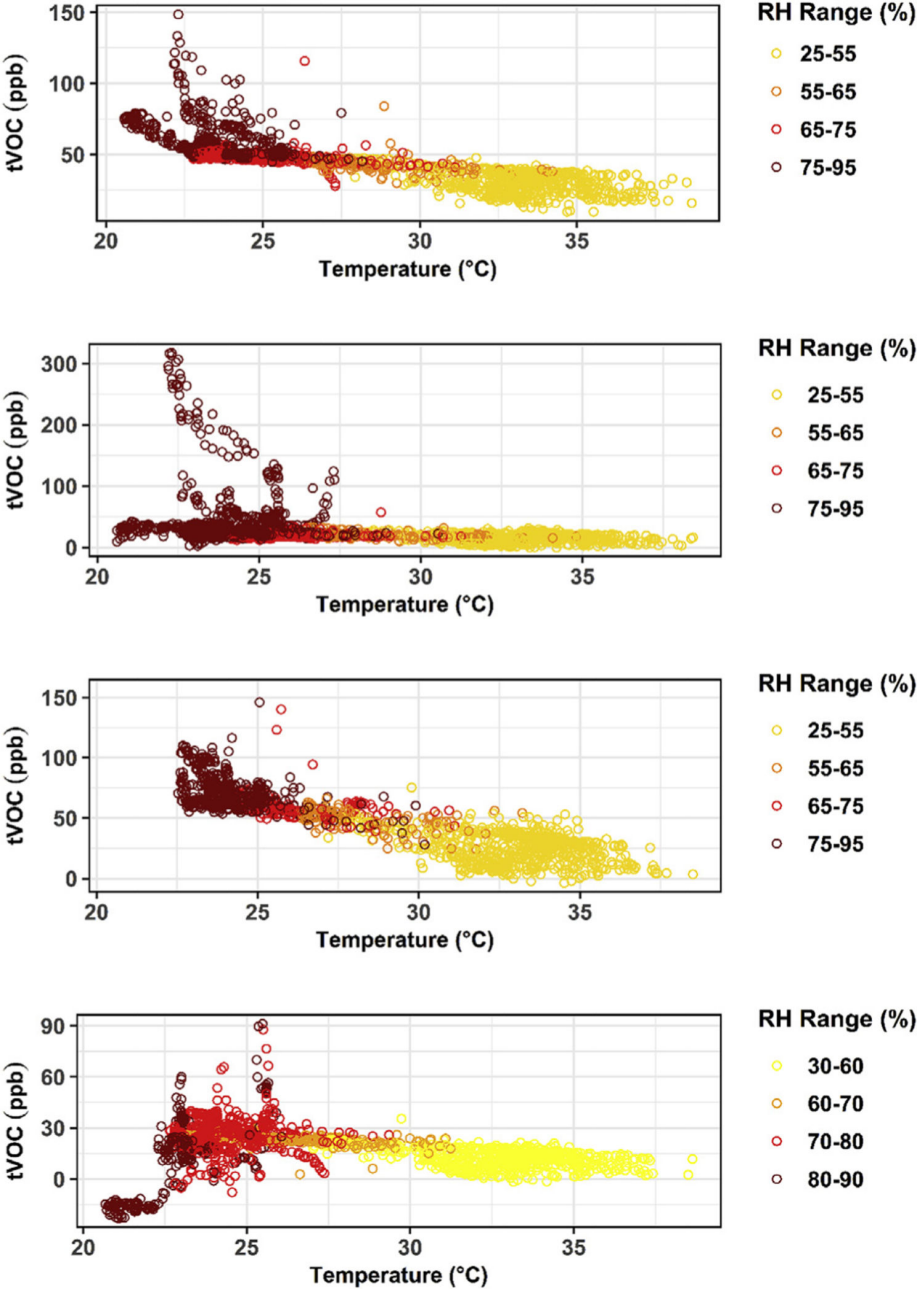
Variable low-cost tVOC replicate sensor response resulting from influence of both temperature and RH factors.

**Fig. 3.**
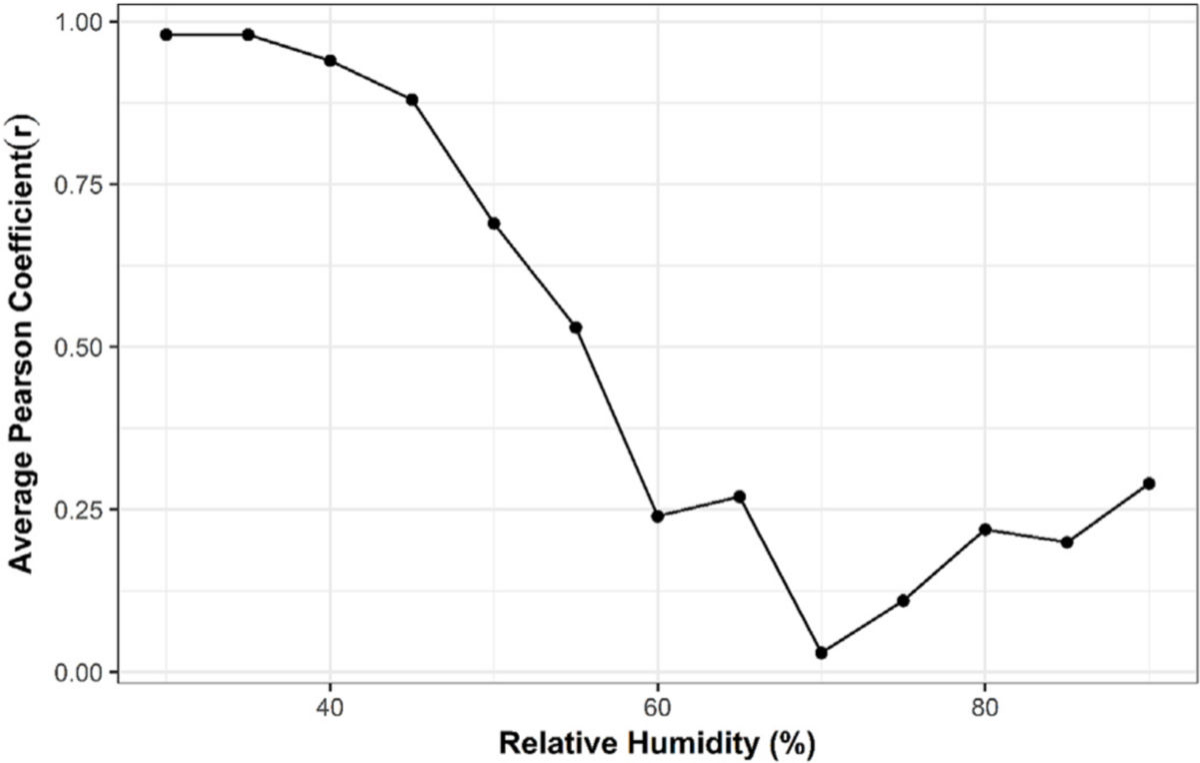
The loss of Pearson correlation between replicate tVOC sensors as a function of increasing RH conditions.

## HIGHLIGHTS

- Examples of significant variability in collocated low cost replicate sensor response were shown for a PM and tVOC device.
- Environmental factors such as RH and temperature were shown to influence the tVOC response.
- Factors influencing all of the individual response deviations in the sensor examples could not be identified.
- End users are encouraged to carefully examine low cost sensor data quality.
